# Dental remineralization via poly(amido amine) and restorative materials containing calcium phosphate nanoparticles

**DOI:** 10.1038/s41368-019-0048-z

**Published:** 2019-05-09

**Authors:** Kunneng Liang, Suping Wang, Siying Tao, Shimeng Xiao, Han Zhou, Ping Wang, Lei Cheng, Xuedong Zhou, Michael D. Weir, Thomas W. Oates, Jiyao Li, Hockin H. K. Xu

**Affiliations:** 1 State Key Laboratory of Oral Diseases & National Clinical Research Center for Oral Diseases & Department of Cariology and Endodontics, West China Hospital of Stomatology, Sichuan University, Chengdu, China; 2 Department of Advanced Oral Sciences and Therapeutics, University of Maryland School of Dentistry, Baltimore, MD USA; 3 Department of Operative Dentistry and Endodontics & Stomatology Center, The First Affiliated Hospital of Zhengzhou University, Zhengzhou, China; 4 Center for Stem Cell Biology & Regenerative Medicine, University of Maryland School of Medicine, Baltimore, MD USA; 5 Marlene and Stewart Greenebaum Cancer Center, University of Maryland School of Medicine, Baltimore, MD USA

**Keywords:** Dental biomaterials, Calcium-based cement, Biochemistry

## Abstract

Tooth decay is prevalent, and secondary caries causes restoration failures, both of which are related to demineralization. There is an urgent need to develop new therapeutic materials with remineralization functions. This article represents the first review on the cutting edge research of poly(amido amine) (PAMAM) in combination with nanoparticles of amorphous calcium phosphate (NACP). PAMAM was excellent nucleation template, and could absorb calcium (Ca) and phosphate (P) ions via its functional groups to activate remineralization. NACP composite and adhesive showed acid-neutralization and Ca and P ion release capabilities. PAMAM+NACP together showed synergistic effects and produced triple benefits: excellent nucleation templates, superior acid-neutralization, and ions release. Therefore, the PAMAM+NACP strategy possessed much greater remineralization capacity than using PAMAM or NACP alone. PAMAM+NACP achieved dentin remineralization even in an acidic solution without any initial Ca and P ions. Besides, the long-term remineralization capability of PAMAM+NACP was established. After prolonged fluid challenge, the immersed PAMAM with the recharged NACP still induced effective dentin mineral regeneration. Furthermore, the hardness of pre-demineralized dentin was increased back to that of healthy dentin, indicating a complete remineralization. Therefore, the novel PAMAM+NACP approach is promising to provide long-term therapeutic effects including tooth remineralization, hardness increase, and caries-inhibition capabilities.

## Introduction

Tooth decay is the most common oral disease, which is mainly due to the demineralization of dental hard tissues^1–4^. In the oral environment, there is a physiological equilibrium between the demineralization and remineralization^5^. However, when the acids from cariogenic bacteria increase, the balance tilts toward to demineralization and net mineral loss, eventually leading to caries^3,6^. Therefore, it would be highly beneficial to develop a new generation of bioactive and therapeutic dental materials that have functionalities to suppress demineralization and promote remineralization.

Hydroxyapatite (HA) is the main ingredient of tooth minerals^7^. When in contact with saliva, the following reaction occurs^8^:

$$\begin{array}{l}{\mathrm{Ca}}_{10}({\mathrm{PO}}_{\mathrm{4}})_6({\mathrm{OH}})_2 \rightleftarrows 10{\mathrm{Ca}}^{2 + } + {\mathrm{6PO}}_{\mathrm{4}}^{3 - } + 2{\mathrm{OH}} - \\ {\mathrm{Remineralization}}({\mathrm{Precipitation}}) \rightleftarrows {\mathrm{Demineralization}}({\mathrm{Dissolution}})\end{array}$$

Hence, promoting tooth remineralization requires: (1) increasing the calcium (Ca) and phosphate (P) ion concentrations; and (2) raising the local pH where remineralization is needed.

The demineralized dentin is mainly composed of collagen fibrils, which only have a weak nucleation template capability^9,10^. Therefore, it is important to supply nucleation template materials for the demineralized dentin to attract Ca and P ions, thereby activating and accelerating the remineralization process. Previously published meritorious reviews on dental remineralization are available^5,11–15^. The present article represents the first review that focuses on remineralization via novel poly(amido amine) (PAMAM) dendrimers having great nucleation capability, together with a new generation of bioactive resins with acid-neutralization, as well as Ca and P ion recharge and re-release functions.

## Using PAMAM as nucleation templates for dental remineralization

Nature is rich with examples of biomineralized hard tissues possessing outstanding mechanical properties and complex morphologies that emerge from their hierarchically arranged structures^16^. Dentin and enamel are two of those examples. Dental caries and erosion are common oral diseases. To promote the remineralization of dentin or enamel via coating remineralization materials on the tooth is a non-invasive therapeutic technique in clinical practice^3^. Dentin is composed of HA, organic matrix, and water^17^. Collagen fibrils are the main component of the demineralized dentin. Some studies showed that collagen fibrils did not have the ability to induce HA nucleation or growth^18,19^, while other studies indicated that collagen fibrils could initialize nucleation and apatite deposit through its carbonyl and carboxy groups^20,21^. However, the nucleation rate of collagen fibrils is far too slow without the use of nucleation template materials^10^. Non-collagenous proteins (NCPs), although composing of only about 3% of the organic components of dentin, play a vital role in the modulation of the biomineralization process^22–25^. NCPs are regarded as the nucleation templates within the collagen fibrils, which can control the hierarchical growth of HA^26–29^. In mature dentin, however, NCPs lose the ability to initialize remineralization^14^. Thus, the strategy of looking for analog materials that act in the role of NCPs has been studied. Mature enamel consists of ~96% of highly organized hierarchical prisms which are derived from nanorod-like HA crystals, and 4% of organic compounds. Extracellular matrix proteins, of which amelogenin is a well-known example, act as nucleation templates in the biomineralization process of enamel^30–32^. Once mature enamel is demineralized, it cannot regenerate because there are no ameloblasts to secrete amelogenin. Therefore, designing analog materials that play the role of extracellular matrix proteins to acquire the hierarchical structure of enamel is needed to repair the defective enamel.

Mimicking the functions of NCPs, phosphate-based template analogs of matrix proteins were synthesized by immobilizing amorphous calcium phosphate (ACP) to induce apatite nucleation and growth at intrafibrillar locations^33–36^. Polydopamine-induced effective biomimetic remineralization of demineralized dentin^10^. In addition, amelogenin, enamel matrix derivative, and gelatin were applied to initiate the growth of aligned apatite nanocrystals on enamel^37–39^. Furthermore, the disorder–order interplay of elastin-like recombinamers were exploited to enable highly controlled and hierarchical mineralization resembling enamel^40^.

PAMAM dendrimers were investigated as nucleation templating analogs for biomineralization^41^. PAMAM are highly branched polymers with internal cavities and a large number of reactive terminal groups^41^. Several different generations of PAMAM with different structures were synthesized^42^. The first and second generations of PAMAM are linear molecules, while the third and higher generations are sphere molecules with a larger number of functional groups^41^. PAMAM have diversified types of terminal groups, including carboxyl-terminated PAMAM (PAMAM–COOH), hydroxy-terminated PAMAM (PAMAM–OH), amine-terminated PAMAM (PAMAM–NH_2_), and phosphorylated PAMAM (PAMAM–PO_3_H_2_).

**Fig. 1:**
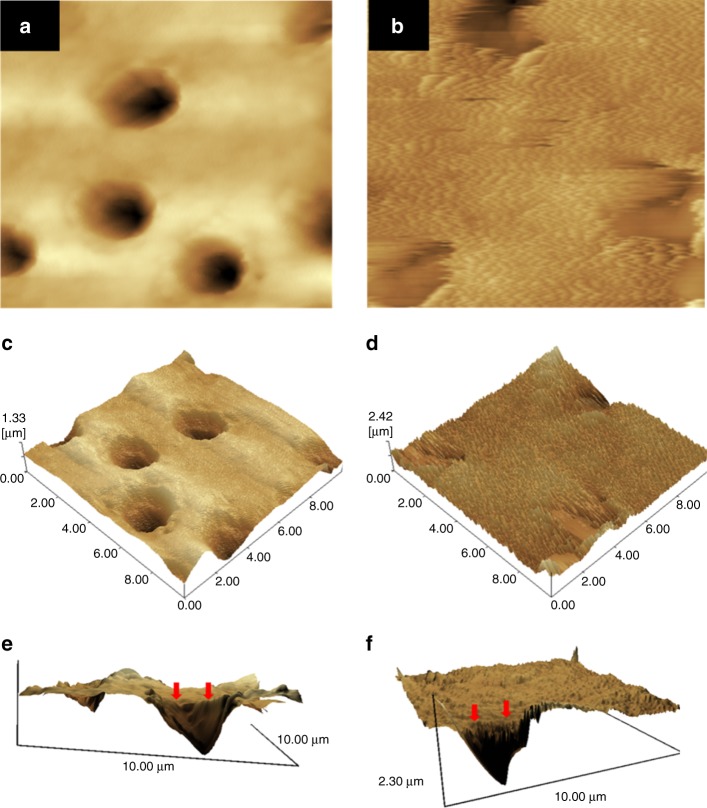
Representative AFM images before and after being immersed in artificial saliva for 4 weeks. AFM images of the demineralized dentin surfaces without treatment (**a**, **c**, **e**) and with the treatment of PAMAM-NH_2_ (**b**, **d**, **f**) after being immersed in artificial saliva for 4 weeks (image area: 10 μm × 10 μm). **a** and **b** are the topographical images. **c** and **d** are the three-dimensional images. **e** and **f** are reconstructed from **a** and **b** by the software SPM-9700, and a horizontal plane of the remineralization surface and longitudinal profile of dentinal tubule were observed. Control showed no mineral regeneration. In contrast, PAMAM–NH_2_ induced large amounts of needle-like minerals in dentin. (Adapted from Liang et al.^50^ with permission.)

**Fig. 2:**
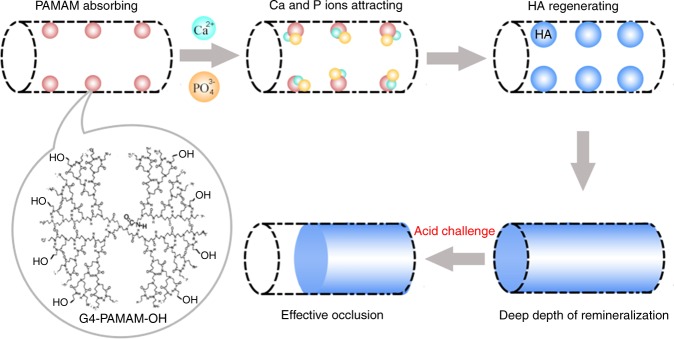
Schematic demonstration of dentinal tubule occlusion via polyhydroxy-terminated PAMAM. First, PAMAM–OH macromolecules bound to collagen fibrils within the tubules through size-exclusion features of collagen fibrils and electrostatic interactions. Then the templates served as a nucleation site to attract calcium ions through calcium complexation by both amide groups and hydroxy groups. Then the calcium ions attracted phosphate ions to form HA. The stable intratubular minerals could resist the acid attack, which ensured that the dentinal tubule occlusion was effective under acidic oral environment. (Adapted from Liang et al.^53^ with permission.)

Studies showed that PAMAM–COOH acted as the organic nucleation template to induce biomimetic new-grown crystals on the demineralized enamel.^43,44^ PAMAM–COOH absorbed Ca and P ions within collagen fibrils to induce intrafibrillar remineralization. The mineralization process initiated by PAMAM–COOH was illustrated and characterized through both in vitro and in vivo experiments^45^. PAMAM–PO_3_H_2_ was absorbed on demineralized enamel surface tightly and produced an enamel prism-like structure, which was also proved in an animal model^46^. PAMAM–PO_3_H_2_ bound tightly to dentin collagen fibrils, and induced effective regeneration of demineralized dentin^47,48^. In addition, PAMAM–NH_2_ induced the regeneration of minerals on both collagen fibril surface and demineralized dentin surface (Fig. 1)^49–52^. Moreover, PAMAM–OH showed a moderate remineralization ability on occluding dentinal tubules^53^. Fig. 2 shows a schematic of effective dentinal tubule occlusion induced by PAMAM–OH^53^. Furthermore, a study concluded that PAMAM–NH_2_ and PAMAM–COOH were more effective than PAMAM–OH on dentin remineralization, because –NH_2_ and –COOH groups had stronger abitlity than –OH group to grab Ca and P ions^54^.

The remineralization mechanism induced by PAMAM was still not clear. Some researchers speculated one possible mechanism: PAMAM–NH_2_ macromolecules bind to dentin collagen fibrils via size-exclusion features and electrostatic interactions of collagen^50^. According to size-exclusion feature theories, molecules with a molecular weight between 6 and 40 kDa can be retained within the dentin collagen fibrils^55^. On the other hand, the positively or negatively charged groups of PAMAM–NH_2_ macromolecules help themselves to bind to the charged sites on collagen fibrils via electrostatic interactions^50^. The immobilized PAMAM–NH_2_ macromolecules then play the role of nucleation templates, mimic the functions of organic matrix proteins, thus initializing and accelerating the remineralization process. To date, besides the mechanism of using PAMAM as nucleation templates for remineralization, there have been no reports on other possible remineralization mechanisms via PAMAM. Further study is needed to investigate other remineralization mechanisms via PAMAM.

## Dental composites and adhesives containing calcium phosphate nanoparticles with remineralization capability

Resin composites are popular due to their excellent esthetics, direct-filling capability and enhanced performance^56–61^. However, the demineralization at the restoration margins due to biofilm acids often leads to secondary caries, which is the main reason for restoration failures^62,63^. To address this problem, remineralization ingredients were incorporated into composites^64–70^. Since fluoride is widely used for dental remineralization, fluoride-releasing features were incorporated in the development of bioactive dental composites with remineralization capabilities. Fluorine (F) ions can be incorporated into tooth minerals, leading to fluoroapatite or F-enriched HA, both having a reduced solubility. Hence, F-releasing materials could help inhibit demineralization and facilitate remineralization. Recently, calcium fluoride (CaF_2_) nanoparticles were developed via a spray-drying system^71^. The CaF_2_ nanocomposite showed high F ion release and good mechanical properties, which may promote remineralization and reduce secondary caries^72,73^. However, the opacity of the CaF_2_ nanocomposite will hinder uses where a high level of esthetics is desired, especially when the nano-CaF_2_ filler level is relatively high^72,73^.

Another important approach was to develop calcium phosphate (CaP) resin composites^68,74–77^. Traditional CaP composites, containing CaP fillers with particle sizes of ~1–55 μm, released high levels of Ca and P ions, and remineralized tooth lesions in vitro^74–76^. However, their mechanical properties were too weak for bulk filling applications. Hence, one study developed whisker-reinforced CaP composite with improved mechanical properties^78^. In another study, a barium-glass filler was incorporated into a nanocomposite containing ACP, which yielded an increase in mechanical properties, with no negative influence on ions release^79^. However, the mechanical properties of these traditional CaP composites were still weak, with flexural strength being only about half that of the unfilled resin^74–76^.

**Fig. 3:**
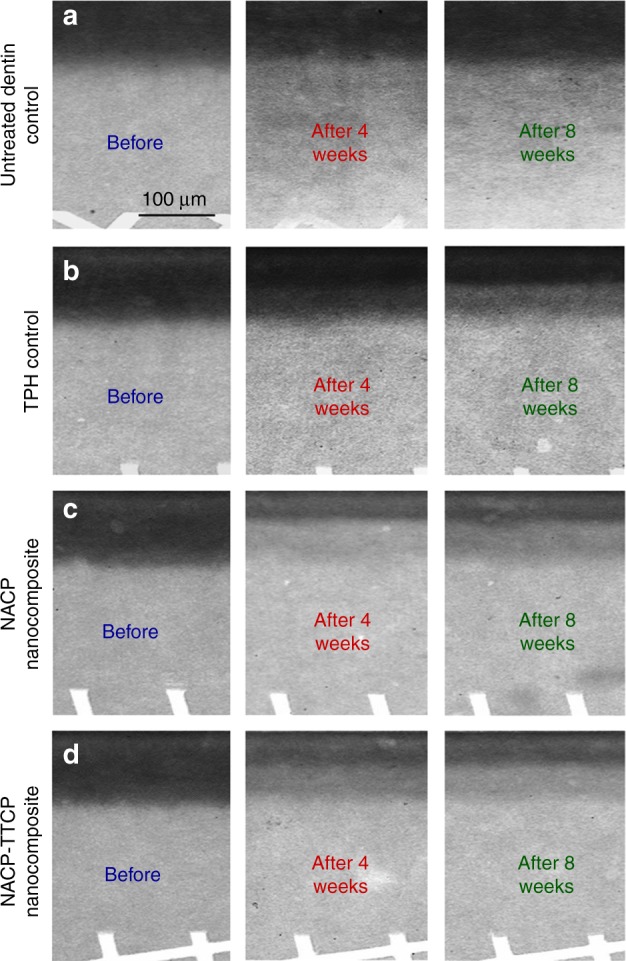
Representative microradiographs of dentin lesions before and after the cyclic demineralization/remineralization regimen. The left column, before, refers to the initial dentin demineralization created in the acidic solution. The middle column is after 4 weeks of the cyclic demineralization/remineralization regimen. The right column is after 8 weeks of the cyclic demineralization/remineralization regimen. NACP composite achieved a much greater remineralization than a commercial control composite. (Adapted from Weir et al.^89^ with permission.)

More recently, nanoparticles of ACP (NACP) with particle sizes of about 100 nm were synthesized^80–86^. The NACP composite released supersaturating levels of Ca and P ions, while possessing flexural strength and elastic modules two-fold those of traditional CaP composites^80^. The NACP composite was “smart”, which greatly increased its ions release in an acidic condition, and rapidly neutralized acids, raising a cariogenic pH of 4 to a safe pH of above 5.5^87^. Indeed, NACP composite was shown to promote tooth remineralization^88–90^. In an in vitro study, NACP composite remineralized artificial enamel lesions in a cyclic demineralization/remineralization regimen, inducing a remineralization that was four-fold that of a commercial F-releasing composite^88^. In another study, NACP composite facilitated dentin remineralization, achieving a remineralization much higher than that of a commercial nanocomposite (Fig. 3)^89^. In a human in situ model, 25 volunteers wore palatal devices containing enamel slabs with cavities restored with NACP or control composite. The NACP composite showed great caries-inhibition under biofilm acids in the oral environment, with enamel mineral loss that was only 1/3 that of a control composite^90^.

Besides composites, NACP were also incorporated into adhesives^91–93^. Due to the small size of NACP, the nanoparticles flowed with the adhesive into dentinal tubules to form bioactive resin tags^91–93^. The NACP adhesive infiltrated into the demineralized dentin collagen matrix to form a hybrid layer (HL)^94–97^. Mineral crystals can protect the HL from external risk factors. Therefore, promoting the remineralization of HL is a promising strategy to prolong the longevity of the resin–dentin bonds^98–100^. The NACP adhesive inhibited dentin demineralization by neutralizing the acids, and promoted the dentin remineralization by releasing large amounts of Ca and P ions^101^. Therefore, the NACP adhesive is promising to help prolong the resin–dentin-bonded interface, which warrants further investigation.

## Remineralization via the combination of PAMAM with NACP

As we stated above, inducing dentin remineralization requires: (1) providing nucleation templates; (2) increasing the Ca and P ion concentrations; and (3) raising the local pH. PAMAM is an excellent nucleation template and can activate the remineralization process by absorbing Ca and P ions^47,50,51,53^. However, PAMAM cannot neutralize acids nor release Ca and P ions. In contrast, NACP can rapidly raise the local pH via neutralizing acids, and release large amounts of Ca and P ions^87–89^. Therefore, it would be advantageous to combine NACP with PAMAM to achieve triple benefits: excellent nucleation template, high level of Ca and P ions release, and strong acid-neutralization capacity. This unique synergistic effect between PAMAM and NACP is a major advantage of the PAMAM+NACP approach, when compared with other remineralization materials.

In a recent study^102^, the combination of PAMAM and NACP composite was used to induce dentin remineralization. A PAMAM solution was prepared by dissolving PAMAM powder (50 mg) in deionized water (50 mL) to achieve a concentration of 1 mg•mL^–1^. NACP composite was fabricated by incorporating 30% NACP and 40% glass particles into a resin. Demineralized dentin samples were: (1) treated with deionized water, (2) coated with PAMAM solution, (3) placed in contact with NACP composite, and (4) coated with PAMAM and then placed with NACP composite. Each day, the four groups were immersed in 1 mL of pH 7 artificial saliva for 23 h, and then in 1 mL of pH 4 lactic acid solution for 1 h at 37 °C. This pH cycling was repeated for 21 days. PAMAM and NACP composite each alone facilitated moderate remineralization. In contrast, the PAMAM+NACP combination induced much greater remineralization, with large amounts of needle-like mineral crystals in dentin surfaces and in dentinal tubules. In addition, the PAMAM+NACP combination increased the hardness of the pre-demineralized dentin back to that of healthy dentin, indicating a complete remineralization of the pre-demineralized dentin.

**Fig. 4:**
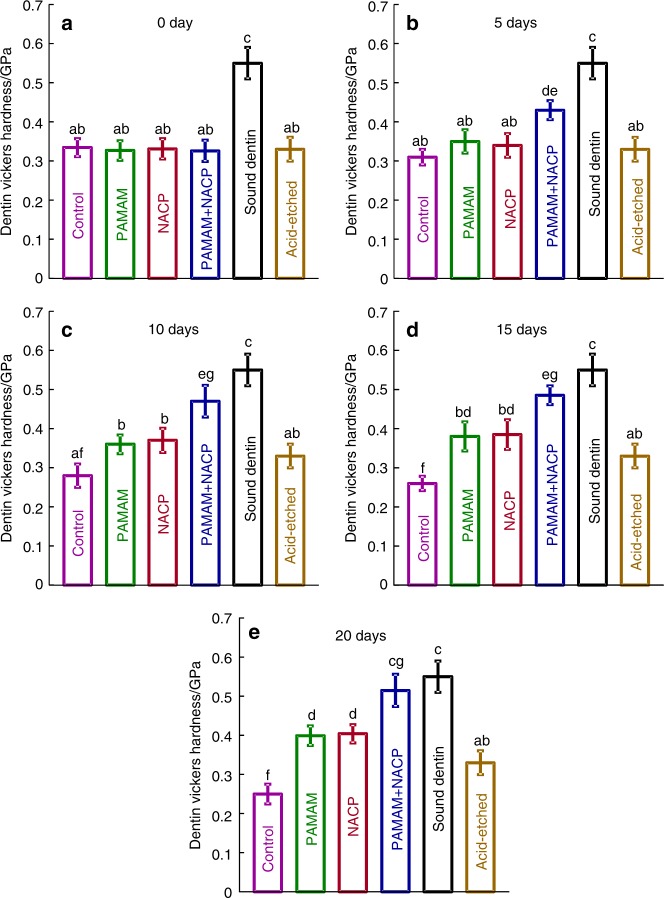
Hardness of demineralized dentin after 0, 5, 10, 15 and 20 days of artificial saliva/lactic acid regimen for: Control group, PAMAM group, NACP group, and PAMAM+NACP group. Hardness of healthy dentin and acid-etched dentin were also measured as comparative controls. The NACP filler level was 40%. PAMAM and NACP each alone mildly increased the hardness of demineralized dentin. PAMAM+NACP achieved the most dentin remineralization, yielding the greatest increase in dentin hardness, which approached that of healthy dentin (*P* > 0.1). In each plot, dissimilar letters indicate significantly different values (*P* < 0.05). (Adapted from Liang et al.^101^ with permission.)

Besides composite, PAMAM+NACP bonding agent was also investigated for dentin remineralization^101^. A PAMAM solution with a concentration of 10 mg•mL^–1^ was prepared, and a bonding agent containing 40% NACP was fabricated. Four groups were examined: (1) control dentin, (2) dentin coated with PAMAM, (3) dentin in contact with NACP bonding agent, and (4) dentin with PAMAM+NACP bonding agent. The four groups were treated with a cyclic artificial saliva/lactic acid regimen for 10 days. PAMAM induced mild remineralization via its nucleation template function. NACP bonding agent neutralized acids to inhibit demineralization in lactic acid, and released Ca and P ions in artificial saliva, producing a moderate remineralization. In contrast, the PAMAM+NACP bonding agent combination induced the greatest dentin remineralization, with the most mineral regeneration and the greatest increase in dentin hardness (Fig. 4)^101^. Therefore, the novel PAMAM+NACP combination strategy showed excellent remineralization capability, and is promising for a wide range of dental applications to prolong the resin–dentin bonds, protect dental hard tissues, and inhibit secondary caries. Further studies using human in situ models are needed to investigate the efficacy and mechanisms of PAMAM+NACP in vivo.

## Remineralization in an extreme acid challenge environment via PAMAM+NACP

Saliva plays an important role for healthy individuals to inhibit caries^103–106^. With a constant flow, saliva helps remove bacteria via swallowing. Saliva can also neutralize biofilm acids by its strong buffer capability^103–106^. In addition, the high concentrations of Ca and P ions in saliva help promote remineralization. However, many factors can cause saliva reduction, such as Sjögren’s syndrome, nutrition deficiency, salivary dysfunction, certain medicines and systemic diseases, etc.^103–106^. Indeed, about one-third of the population reports dry mouth^104^.

For individuals with saliva reduction, the saliva flow could drop to about 15–33% of its normal level^107^. This means much less saliva to penetrate into bacteria biofilms to neutralize acids and provide Ca and P ions. This leads to an acidic and Ca and P ion-deficient microenvironment around the tooth, thus increasing the caries risk. Saliva reduction occurs often in seniors. Indeed, in the USA, root caries increased with aging, from 7% in young generation, to 56% among people of ≥75 years of age^108^. Furthermore, extreme saliva reduction often occurs in patients with head and neck cancers taking radiation therapy, and the extreme acidic and ion-deficient oral environment often causes rampant radiation caries^109–111^. Traditional caries-inhibition strategies are ineffective for patients with severe saliva reduction. For example, in an in vivo study, casein phosphopeptide ACP (CPP-ACP) failed to prevent radiation caries^112^. Therefore, there is a great need for new remineralization strategies that are effective in an acidic and Ca and P ion-deficient environment.

**Fig. 5:**
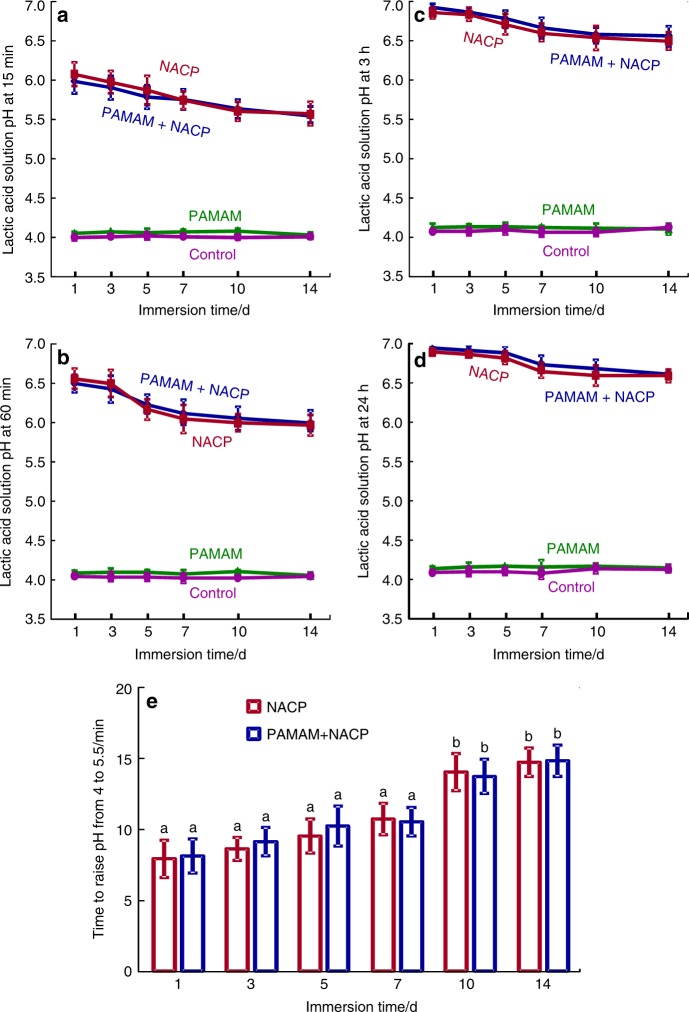
Acid neutralization. The lactic acid pH was measured after the sample was immersed for: (**a**) 15 min, (**b**) 60 min, (**c**) 3 h, and (**d**) 24 h (mean ± SD; *n* = 6). This was repeated for 14 days. (**e**) The time it took to raise the pH from 4 to 5.5. NACP and PAMAM+NACP neutralized the acid and increased the pH. The acid neutralization ability of NACP adhesive decreased over time due to the use of a fresh acid solution each day. PAMAM and control groups showed no acid neutralization, with pH staying near 4. (Adapted from Liang et al.^114^ with permission.)

**Fig. 6:**
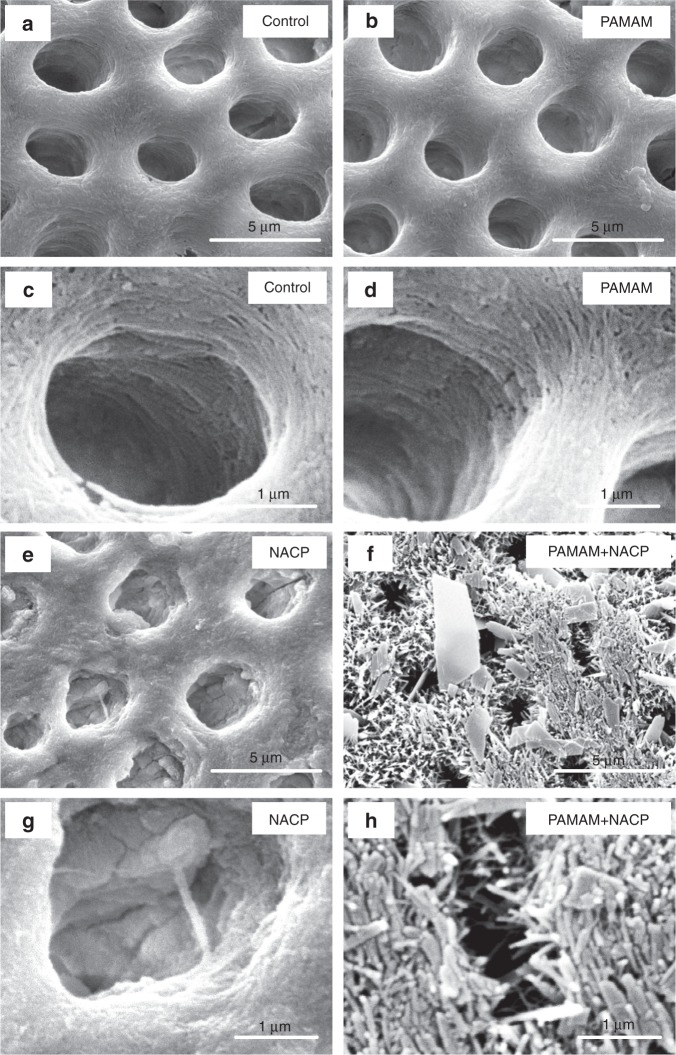
Representative SEM images of dentin perpendicular to tubule axis after 21 days lactic acid immersion. Representative SEM images of demineralized dentin perpendicular to tubule axis after 21 days lactic acid immersion: (**a**, **c**) control, (**b**, **d**) PAMAM, (**e**, **g**) NACP, and (**f**, **h**) PAMAM+NACP. Exposed collagen fibrils were observed in (**c** and **d**). Images (**e**–**h**) showed regenerated minerals in demineralized dentin. For PAMAM+NACP group in image (**h**), numerous needle-like minerals precipitated in dentin and occluded the tubules. (Adapted from Liang et al. ^113^ with permission.)

For the first time, in a pH 4 lactic acid solution without any initial Ca and P ions, the combination of PAMAM and NACP method successfully promoted dentin remineralization^113,114^. Demineralized dentin samples with (1) PAMAM, (2) NACP, and (3) PAMAM+NACP were immersed in pH 4 lactic acid solution for 24 h, and the solution was replaced by fresh pH 4 lactic acid solution each day. The 24 h of lactic acid immersion each day was used to simulate the most severely challenging oral condition for patients with the most severe saliva reduction. This was repeated for 21 days or 14 days, for NACP composite and NACP adhesive, respectively. The results showed that PAMAM did not cause any positive effects on protecting dentin structure in the lactic acid. This was because PAMAM could not fulfill its nucleation template function in an environment without any Ca and P ions. NACP prevented further dentin demineralization by neutralizing acids and releasing Ca and P ions. Remarkably, the PAMAM+NACP method achieved dentin remineralization even in the lactic acid, due to the synergy between PAMAM and NACP: NACP rapidly raised the pH of lactic acid to above 6 and released high levels of Ca and P ions, turning the lactic acid to a remineralization solution (Fig. 5)^114^. Then, PAMAM employed the Ca and P ions to facilitate remineralization, thus regenerating the minerals in the demineralized dentin (Fig. 6)^113^. The PAMAM+NACP composite increased the hardness of the demineralized dentin from 0.32 to 0.43 GPa at 7 days, to 0.49 GPa at 14 days, and to 0.53 GPa at 21 days, which approached that of healthy dentin^113^. In addition, the combination of PAMAM and NACP adhesive increased the dentin hardness from 0.31 to 0.56 GPa at 28 days, which was close to that of healthy dentin^114^. Therefore, the PAMAM+NACP synergy is promising to protect the teeth for seniors with gingiva recession and root caries, individuals with dry mouth, and especially those with severe saliva reduction and radiation caries. Further study should investigate the PAMAM+NACP remineralization in situ for the individuals with reduced saliva in the oral environment.

## PAMAM+NACP induced long-term dentin remineralization

Although the PAMAM+NACP method exhibited excellent remineralization capability, the ions release and acid-neutralization capabilities of NACP decreased with time^101,102,113,114^. In addition, with the fluids flow in the oral environment, PAMAM may be detached from the dental hard tissues with time, thus removing the nucleation templates. The process of secondary caries usually takes more than a year to form^115,116^. Therefore, the remineralization capability of PAMAM+NACP needs to be long-term.

Rechargeable NACP composite was developed using a resin of ethoxylated bisphenol A dimethacrylate (EBPADMA) and pyromellitic glycerol dimethacrylate (PMGDM), which was referred to as EBPM^117,118^. This EBPM+NACP composite could be repeatedly recharged with Ca and P ions, and then re-released these ions^117,118^. The mechanisms of the recharge process likely involves: (1) the Ca ion chelating capacity of PMGDM, and (2) the Ca and P ion space-occupying effect^119^. In addition, a rechargeable NACP adhesive was also developed using a resin of PMGDM, EBPADMA, 2-hydroxyethyl methacrylate (HEMA), and bisphenol A glycidyl dimethacrylate (BisGMA), which was referred to as PEHB^120^. After being recharged, the ion-exhausted PEHB+NACP adhesive continuously re-released Ca and P ions for 2–3 weeks^121^. Therefore, the rechargeable EBPM+NACP composite and PEHB+NACP adhesive provided the possibility for long-term remineralization.

**Fig. 7:**
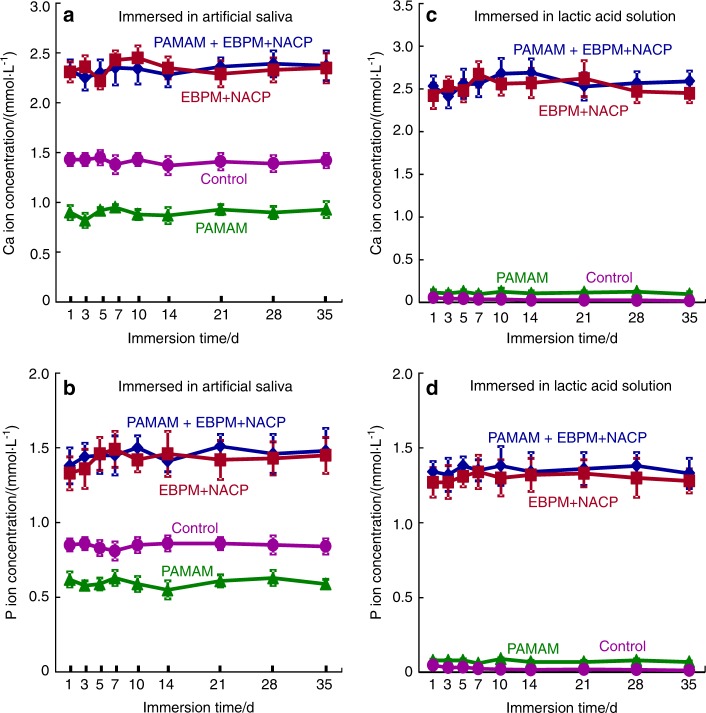
Ion concentrations (mean ± SD; *n* = 6). (**a**, **b**) Ca and P ion concentrations of artificial saliva and (**c**, **d**) lactic acid. EBPM+NACP group and PAMAM+EBPM+NACP group had higher Ca and P concentrations than control and PAMAM groups. PAMAM group had lower ion concentrations in artificial saliva than control group, indicating that PAMAM promoted Ca and P ion precipitation in demineralized dentin during artificial saliva immersion. For EBPM+NACP and PAMAM+EBPM+NACP groups, the ion concentrations did not decrease with repeated recharge, even though a fresh solution was used to immerse the samples every day. (Adapted from Liang et al.^122^ with permission.)

**Fig. 8:**
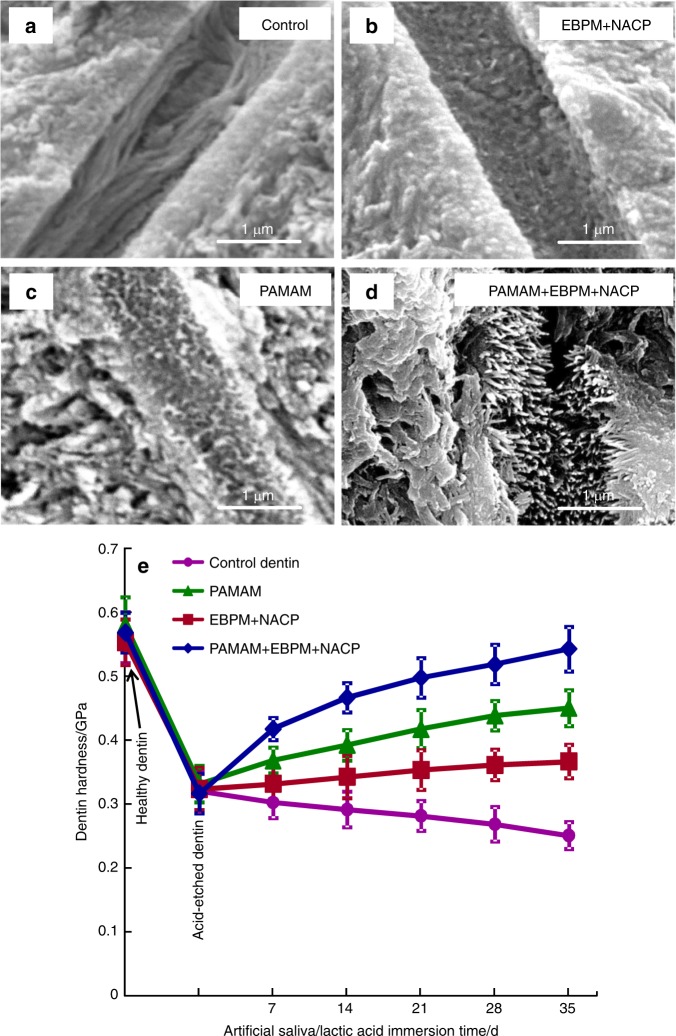
Representative SEM micrographs of dentin cross-sections parallel to the dentinal tubule after 35 days cyclic artificial saliva/lactic acid regimen. SEM micrographs of demineralized dentin cross-sections parallel to the dentinal tubule axis after 35 days cyclic artificial saliva/lactic acid regimen: (**a**) control group, (**b**) EBPM+NACP, (**c**) PAMAM group, and (**d**) PAMAM+EBPM+NACP. (**e**) Dentin hardness. The hardness of healthy dentin, dentin after being acid-etched, and demineralized dentin after 7, 14, 21, 28, and 35 days of artificial saliva/lactic acid cyclic treatments for: control, PAMAM group, EBPM+NACP group, and PAMAM+EBPM+NACP group (mean ± SD; *n* = 6). Images were taken on dentin cross-sections in a subsurface region of 2–30 μm beneath the surface. In (**a**), tubules were full of exposed collagen fibrils. **b** and **c** shows mineral deposit in tubules due to remineralization. In (**d**), tubules were completely occluded by dense minerals. (**e**) PAMAM+NACP returned the hardness of demineralized dentin to the level of healthy dentin (*P* > 0.1). (Adapted from Liang et al.^122^ with permission.)

A recent study demonstrated the long-term remineralization of PAMAM+NACP for the first time^122^. First, demineralized dentin was coated with PAMAM, and then immersed in a phosphate-buffered saline (PBS, pH 7.4) with a constant severe shaking for 72 days (referred to as the immersed-PAMAM-coated dentin). This action simulated saliva flow and drinking which could potentially detached the coated PAMAM. Separately, EBPM+NACP composite was immersed in a pH 4 lactic acid solution for 72 days to completely exhaust the Ca and P ion release. Then, the ion-exhausted EBPM+NACP composite was recharged with a Ca and P ion solution simulating a mouthwash. The EBPM+NACP composite was then placed in contact with the immersed-PAMAM-coated dentin, and the construct was treated with a cyclic remineralization/demineralization regimen for 35 days. The results showed that most of the PAMAM molecules were still attached on the demineralized dentin after 72 days of fluid shaking challenge. The recharged EBPM+NACP composites re-released Ca and P ions, and the ion-release and acid-neutralization capacity did not decrease with increasing the number of release-recharge cycles (Fig. 7). As a result, the immersed-PAMAM and recharged EBPM+NACP composite achieved triple benefits: stable nucleation templates, constant Ca and P ion release, and continuous acid-neutralization. The combination yielded the greatest mineral precipitation both in the dentinal tubules and on the dentin surface (Fig. 8)^122^. In addition, the hardness of the pre-demineralized dentin was increased to that of healthy dentin. Therefore, the PAMAM+NACP strategy is promising to provide long-term tooth-protection and caries-inhibition effects. Further studies are needed to investigate the remineralization durability of PAMAM+NACP for longer terms, for example, for 6 months and 12 months. In addition, applying the PAMAM solution before placing the NACP composite and adhesive may affect the bonding strength and decrease the long-term remineralization effect. Further study is needed to determine whether the application of PAMAM would negatively affect the bonding strength of NACP composite and adhesive.

## Conclusions

This article represents the first review on the combination of PAMAM with calcium phosphate nanoparticles to achieve bioactive and therapeutic properties to inhibit caries. PAMAM and NACP together achieved synergy and yielded triple benefits: excellent nucleation template, superior acid-neutralization, and Ca and P ion release. Even in an acidic pH 4 solution without any initial Ca and P ions, or after a long period of fluid flow challenge, the novel PAMAM+NACP method still induced complete dentin remineralization and increased the hardness of pre-demineralized dentin back to the hardness of healthy dentin. Therefore, the novel PAMAM+NACP strategy is promising to inhibit demineralization and provide long-term remineralization to inhibit caries and protect tooth structures.

## Supplementary information

Figure 1 Permission

Figure 2 Permission

Figure 3 Permission

Figure 4 Permission

Figure 5 Permission

Figure 6 Permission

Figure 7 and 8 Permission
