# Supplementary material for: Dental remineralization via poly(amido amine) and restorative materials containing calcium phosphate nanoparticles
Source: Int J Oral Sci. 2019 May 9;11(2):15. doi: 10.1038/s41368-019-0048-z (PMC6506538; doi:10.1038/s41368-019-0048-z)
Supplement: Supplementary file 2 — Figure 2 Permission [file 41368_2019_48_MOESM2_ESM.docx]

RSC ADV is an open access Journal, and we are the authors of the original paper, therefore, we have the copyright of figure 2.
